# How law legitimized and later recharacterized forced sterilization in Peru: the juridical laundering of biopower

**DOI:** 10.3389/fsoc.2026.1944164

**Published:** 2026-10-28

**Authors:** Andherson Jhordan Aguirre-Lanegra, Jonathan Rafael García Enríquez

**Affiliations:** Universidad Tecnológica del Perú, Lima, Peru

**Keywords:** accountability, biopower, forced sterilization, legal characterization, Peru, reproductive governance, sociology of law, symbolic power of law

## Abstract

Between 1996 and 2000, a Peruvian family planning program sterilized hundreds of thousands of people, most of them poor, rural and Indigenous women, many without free consent. The program operated under the cover of a law, a ministerial resolution, numerical sterilization targets and signed consent forms. Since 1997, Peruvian prosecutors and courts, Congress and international human rights bodies have classified the same facts in contradictory ways, for example as an accident, as ordinary crimes barred by time limits, as a lawful health program and as serious human rights violations. This raises a question for the sociology of law: how do legal documents make state control over reproduction look legitimate, and how do they shape later disputes over it? Existing research explains either how such policies were legitimized or how they were contested, but not the link between the two. To connect them, this article proposes the juridical laundering of biopower, a category with two phases. Biopower here means the state’s power over a population’s life and reproduction. First, legal documents present the state’s intervention as lawful, technical and, when framed as voluntary, consented to. Later, prosecutors, courts and other institutions place the same facts in different legal categories, and the category that prevails determines whether anyone can be held responsible. We test four propositions against the program’s documents and 34 decisions and other official acts issued between 1997 and 2026. The evidence supports three of them: the program’s own documents later served as grounds for not prosecuting its architects, the facts were classified in contradictory ways, and none of its architects was convicted. It is consistent with the fourth, on the conditions for accountability. Classifying the facts as human rights violations led to accountability only when domestic and international institutions agreed on that classification, when a binding court decision enforced it, and when time limits did not bar prosecution. The Peruvian case thus shows how a policy that the State presented as lawful can end in a finding that the State itself violated human rights, while no official is held criminally responsible. We call this outcome deferred impunity.

## 1 Introduction

In the twentieth century, states in several regions sterilized large numbers of people without their free consent, and they often did so through legal instruments (Patel, 2017). Large-scale campaigns have been documented in North America, Latin America and Europe, where hundreds of thousands of Indigenous and racialized people, most of them women, were sterilized in state-sponsored procedures (Rowlands et al., 2025). They include sterilizations under the Third Reich and in several US states, the mass vasectomies carried out in India during the state of emergency declared in 1975, and programs aimed at ethnic minorities or prisoners in Japan, Sweden, Czechoslovakia, Mexico and Puerto Rico (Silva Santisteban, 2021). Such policies were frequently presented as beneficial: the Peruvian government held that sterilizing Indigenous women would reduce their poverty, and Czechoslovak directives described sterilization as a preventive social measure (Rowlands et al., 2025). Peru’s National Reproductive Health and Family Planning Program of 1996–2000 belongs to this group. Its sterilizations were carried out under the cover of a law, a ministerial resolution, numerical sterilization targets, protocols and consent forms that gave them an appearance of legitimacy.

By legal form we mean the set of legally valid instruments through which a policy is authorized and recorded, and in the Peruvian case it mattered in two ways. At the time, it made an intervention in the reproduction of poor, rural and Indigenous women look like a lawful health policy to which they had consented. Afterwards, it shaped the dispute over what that intervention had been. That dispute concerns the legal characterization of the facts, by which we mean the legal category in which an official act places them, such as a fortuitous event, an ordinary offense or a serious human rights violation. Between 1997 and 2026, prosecutors, Congress, the executive, domestic courts and three international bodies gave the same facts incompatible legal characterizations. They are incompatible because each excludes the others: a death cannot be both an accident for which no one is responsible and a human rights violation for which the State is responsible. The three international bodies are the Inter-American Commission on Human Rights, the Inter-American Court of Human Rights and the United Nations Committee on the Elimination of Discrimination against Women (hereinafter the Inter-American Commission, the Inter-American Court and the CEDAW Committee). We use the term official bodies for these and other domestic and international institutions whose acts have legal effect. The law, the program’s purpose and the consent forms reappeared among the reasons given for not prosecuting those who had designed the program (Inter-American Court, 2025, para. 93; Novoa Curich, 2014). The Inter-American Court has since found that the sterilizations were part of a State policy rather than a series of isolated abuses (Inter-American Court, 2025, paras. 152–153), so their legal history is a dispute over what that policy was.

Two bodies of theory help explain how legal documents can make an intervention look lawful at the time and later shape the dispute over what it was. The first concerns power over life. Foucault uses the term biopower for the form of power that takes the life of a population as its object. It fosters the life of some and withholds that support from others, to the point of letting them die, and it acts on processes such as fertility through forecasts, statistics and overall measures (Foucault, 1978, 2003). Racism, in his account, divides the population between those who must live and those who must die (Foucault, 2003). Mbembe extends this analysis to sovereignty understood as the capacity to decide who may live and who must die, and traces the history of this power through the plantation and the colony (Mbembe, 2003). The concept of reproductive governance brings these ideas to the level of policy, naming the laws, incentives, coercion and moral campaigns through which states and other actors control reproductive behavior (Morgan and Roberts, 2012). The second body of theory is the sociology of law, which explains how legal form legitimizes power. For Bourdieu (1987), the juridical field is the social space in which judges, lawyers, legislators and other legal actors compete over the right to say what the law is. García Villegas (2014) shows that norms can legitimize power even when they are not enforced, a capacity he calls the symbolic efficacy of law.

Research on the Peruvian program has developed along several lines. A first line documented the facts and framed them as human rights violations. Tamayo’s (1999) report gathered testimonies and official documents showing numerical targets, quotas and practices contrary to informed consent. Getgen (2009) argued that the exclusion of the sterilizations from the report of the Truth and Reconciliation Commission removed State responsibility from the official record of the period. The Inter-American Commission (2003), the CEDAW Committee (2024) and the Inter-American Court (2025) later recognized the responsibility of the State, and their decisions are primary sources for this article.

A second line explained the program through population policy and political economy. Boesten (2007) read it as a tension between free choice and poverty alleviation. Ewig showed how the language of reproductive rights adopted after the Cairo and Beijing conferences was used to legitimize demographic and economic goals, and how the program fit into a neoliberal reform of the health sector (Ewig, 2006, 2010, 2014). Rousseau (2007) placed it within 15 years of reproductive health reforms that only partly corrected women’s unequal access to family planning, reproductive rights and maternal care. Coe (2004) showed that its demographic approach, built on numerical targets, was followed after 2001 by conservative policies that restricted reproductive choice from the opposite direction.

A third line studied the experience and the memory of those affected. Chaparro-Buitrago (2022) showed that *campesina* women in Cajamarca describe the harm as a loss of strength rather than as infertility, and with Freeman she used the reproductive justice framework to broaden the range of harms considered (Chaparro-Buitrago and Freeman, 2023). Molina Serra (2017) analyzed the public narratives about the sterilizations, and Ballón (2014) brought together analyses of the case in a collective volume on its memory. Ruiz Alvarado (2024) examined why the registry of victims and the reform of the reparations law have not advanced. Carranza Ko (2020) and Silva Santisteban (2021) interpreted the program as genocide, a reading that De La Cruz Huamán (2018) partly disputes by reporting that most research finds socioeconomic and ethnic discrimination rather than genocide. Studies in medical ethics examined the failures of informed consent (Gianella, 2013; Málaga, 2013).

These studies established what happened, why it happened and with what consequences. Silva Santisteban (2021), moreover, had already read the program in biopolitical terms, as a form of reproductive control linked to patriarchy. Less research has examined the role that law played, both in the program and in its aftermath. Two questions therefore remain open: how legal form made the program appear lawful, technical and consented to while it was carried out, and how the same legal form shaped the characterizations that official bodies gave the facts in the following decades. Studies of power over life have seldom examined how legal documents make a policy look legitimate, and the sociology of law has seldom examined how states intervene in reproductive matters. In short, no existing concept connects two things: how law makes an intervention look legitimate while it is taking place, and how official bodies classify the same facts years later. This article addresses that gap.

This article proposes an analytical category, the juridical laundering of biopower, specifies the conditions under which it applies, and illustrates it with the Peruvian program of 1996–2000 and the legal trajectory of its facts between 1997 and 2026. The category describes a process that works through legal documents and unfolds in two phases. Legally valid instruments first present a state intervention in the life, health or reproduction of a population as lawful, technical and, where it is framed as voluntary, consented to. Later, official bodies of the same juridical field assign successive and incompatible legal characterizations to that intervention, and the characterization that prevails at any given moment determines whether responsibility can be attributed. We speak of laundering by analogy, because legal form gives a lawful appearance to an intervention that would be contested if it were described openly. Our thesis is that the legal documents that make such an intervention look legitimate keep having effects after the intervention ends: years later, they supply evidence and arguments to those who oppose classifying the facts in a new way. We call this persistence the long temporality of the symbolic efficacy of law. A new legal characterization leads to accountability when three conditions hold: different official bodies agree on the new legal characterization, a binding decision enforces it, and the rules on statutory limitation allow prosecution. Statutory limitation sets the time limits for bringing criminal charges. When these conditions fail, the new characterization leads instead to what we call deferred impunity, a prolonged failure to hold any individual criminally responsible. The category is analytical rather than specific to forced sterilization, since it can apply to any state intervention in life, health or reproduction that meets the conditions set out in Section 4. Nor does it cover every forced sterilization: it applies only to sterilizations carried out under legally valid instruments that later received more than one legal characterization. Our evidence, however, comes from one reproductive program, so the case specifies and illustrates the category but cannot establish how broadly it applies, and the conditions we state remain hypotheses for comparative work.

### 1.1 Sources, method and testable propositions

This is a Hypothesis and Theory article: it builds a category from existing theory and illustrates it with a documented case. The corpus consists of every official act that fixed or disputed the legal characterization of the facts; the same criterion defines membership in the juridical field in Section 3. An act was included if an official body issued it, if it concerned the sterilizations performed under the program or a case arising from them, and if it assigned or disputed a legal category for those facts. Statements by the parties, reports by nongovernmental organizations and press coverage do not count as acts, although we use them as sources where noted below. The corpus comprises 34 acts issued between 1997 and 2026, which Section 6 lists and examines. We identified them mainly in two decisions. The first is the Inter-American Court’s judgment in *Ramos Durand*, the case of Celia Ramos, who died in 1997 after being sterilized under the program (Inter-American Court, 2025, para. 82); it reconstructs the domestic proceedings up to 2025. The second is the CEDAW Committee’s Views, its decision on an individual complaint brought by women sterilized under the program. Further sources are the friendly settlement of the Mestanza case, Law 32107 and the decisions of the Constitutional Court and the Supreme Court of Justice of Peru, both of 2025. The Mestanza case concerned Mamérita Mestanza, who died in 1998 after being sterilized under the program, and it was settled before the Inter-American Commission (Inter-American Commission, 2003, para. 12). We checked the resulting list against the domestic judgments of 2025 and against press coverage of 2024–2026. The first phase is documented from the program’s instruments as recorded by those bodies, from Tamayo’s (1999) documentary report and from the scholarship on the case. Press reports and statements by the victims’ representatives are used only for acts that no official document in our corpus records, and each is identified as such (Páez, 2009; DEMUS et al., 2024). They include the judgment that Victoria Vigo, sterilized without her consent in 1996, obtained against the physician who sterilized her, and several decisions of 2024–2026.

For each act we record the body, the date, the legal category it assigned to the facts, the grounds it gave and its consequence for the attribution of responsibility. We distinguish the findings of courts and treaty bodies from the allegations of the parties and the positions of the State, and we report each in its own voice. By analysis of decisions we mean reading the legal category each act assigned and the reasons it gave, not judging whether the act was legally correct. We consulted sources up to 23 September 2026, and the most recent act examined dates from June 2026. Quotations from Spanish-language sources are given in our translation and marked as such. The argument yields four propositions that can be tested against this record (Table 1).

**Table 1:** Testable propositions, their indicators, the observations that would refute them and the result in the Peruvian case.

Proposition	Indicators in the case	Refuted if	Result in the case
P1. Legitimation and reuse: the instruments that authorized the program present it as lawful, technical and consented to, and the same instruments reappear as grounds in later decisions not to prosecute	The 1995 law and the 1996 ministerial resolution; the targets; the consent manual and forms; the grounds of the dismissals of 2014 and 2016	The instruments presented the program openly as coercive, or later decisions not to prosecute rested on grounds unrelated to them	Supported: the dismissals of January 2014 and December 2016 relied on the law, the program’s purpose, the consent manual and a signed form (Sections 5 and 6)
P2. Conflicting legal characterizations: official bodies assign the same facts successive and incompatible legal characterizations, without a final domestic decision	The characterizations listed in Section 6; the opposite readings of Law 32107 by the Constitutional Court and the Supreme Court in 2025	The facts kept one characterization across bodies, or a final domestic decision settled it	Supported: at least six characterizations between 1997 and 2026 and no final domestic decision (Section 6 and Table 3)
P3. No individual criminal responsibility: no domestic court attributes criminal responsibility to those who designed and directed the program	The outcome of the proceedings against Fujimori and senior officials between 2002 and 2026, including Fujimori’s death in 2024 before trial	A domestic court convicts an official responsible for the program at the level of policy	Supported: no such conviction; the proceedings of 2021–2023 were voided and Fujimori died without trial (Section 6)
P4. Conditions: recharacterization leads to accountability where different official bodies agree on the new legal characterization, a binding decision enforces it, and the rules on statutory limitation allow prosecution, and to deferred impunity where these conditions fail	The international decisions and the Supreme Court’s decision of 2025, compared with the dismissals, the annulment of 2023, its confirmation in 2024 and Law 32107; the cases of Barrios Altos and La Cantuta as a comparison	Accountability followed disagreement among official bodies without a binding decision, or no individual was held responsible where all three conditions held	Consistent but not confirmed: State responsibility followed agreement among official bodies and binding decisions, individual responsibility did not, and Barrios Altos and La Cantuta fit the contrast (Section 7)

## 2 Power over life and reproductive governance

Foucault describes a change in how power relates to life. In his account, the sovereign right “to take life or let live” was replaced by “a power to foster life or disallow it to the point of death” (Foucault, 1978, p. 138). This power developed along two poles: an anatomo-politics that disciplines individual bodies and a biopolitics of the population, which regulates births, mortality, health and longevity (Foucault, 1978). Among the processes that biopolitics takes as its object are “the rate of reproduction, the fertility of a population” (Foucault, 2003, p. 243), and its instruments are “forecasts, statistical estimates, and overall measures” (Foucault, 2003, p. 246). The category proposed in this article has the same object: the life, health or reproduction of a population. A national program with fertility targets and coverage statistics is a biopolitical instrument in this precise sense.

In Foucault’s account, law does not disappear under biopower; it changes function. “[T]he law operates more and more as a norm,” he writes, “and … the judicial institution is increasingly incorporated into a continuum of apparatuses (medical, administrative, and so on) whose functions are for the most part regulatory” (Foucault, 1978, p. 144). Law then ceases to be only a limit that prohibits and punishes and becomes one of the means through which a population is administered. A ministerial resolution that approves a health program, sets its targets and regulates its consent forms is law of this kind, since it works inside the medical and administrative apparatus it regulates.

A power whose aim is to foster life needs a way to justify letting some people die, and Foucault finds it in racism. Racism introduces into the domain of life that power controls “the break between what must live and what must die” (Foucault, 2003, p. 254). The killing it allows need not be direct, since Foucault includes every form of indirect murder, such as exposing people to death, increasing their risk of death, or rejecting them (Foucault, 2003). The Peruvian program distributed its interventions unequally, and they fell mainly on poor, rural and Indigenous women (Inter-American Court, 2025, paras. 52, 152–153). Unsafe surgery under the program also exposed people to death: the Ombudsman’s Office (*Defensoría del Pueblo*) received complaints of 17 deaths, 15 of them women, and the Inter-American Court found that Peru had violated Celia Ramos’s right to life (Inter-American Court, 2025, para. 68; operative paragraph 3).

Mbembe shifts the emphasis from the management of life to power over death. He starts from the premise that the ultimate expression of sovereignty lies in “the power and the capacity to dictate who may live and who must die” (Mbembe, 2003, p. 11). His concern is with forms of sovereignty whose central project is “the generalized instrumentalization of human existence and the material destruction of human bodies and populations” (Mbembe, 2003, p. 14). Following Foucault, he treats racism as the technology that decides who is exposed to death within biopower (Mbembe, 2003). He uses the term necropolitics for the contemporary forms in which life is subjected to the power of death. The concept of biopower, he argues, cannot fully account for these forms, whose history he traces through the plantation and the colony (Mbembe, 2003).

Whether biopolitics and necropolitics are opposed or connected remains an open question. Writing on migration, Estévez (2018) argues that they are mutually constitutive, since the legal devices that manage the lives of asylum seekers operate because a necropolitics has expelled those people from their countries. We extend this argument to reproductive policy. The Peruvian program pursued a health goal for the nation while it prevented particular women from having more children. In the terms of Foucault’s formula, the program disallowed life rather than taking it. Silva Santisteban (2021) reads the program in these biopolitical terms, linking the control of reproduction to patriarchy and describing a biopolitics of control over those considered surplus.

Reproductive governance brings this analysis to the level of a health program. For Morgan and Roberts (2012), it comprises the laws, economic incentives, moral pressure and direct coercion through which varied actors, from state and religious institutions to international lenders, nongovernmental organizations and social movements, seek to shape and monitor how people reproduce. Morgan (2019) notes that the concept arose from Latin American transitions to democracy and to neoliberal economies, both built on a commitment to human rights, in states that privatized public services and made citizens responsible for their own health. El Kotni and Singer (2019) add that the Cairo and Beijing agreements placed reproductive decisions within the realm of human rights. In their account, rights then restructured the mechanisms of reproductive governance, which operates through laws, policies and state programs as well as through clinical care and public messaging. The Peruvian program, which combined the language of reproductive rights with demographic aims, is a case of this restructured governance. The reproductive justice framework widens the view further, since it shows that accounts of forced sterilization in Peru have focused on motherhood and fertility and overlooked other harms (Chaparro-Buitrago and Freeman, 2023).

The Peruvian program did not operate through a suspension of law. Agamben’s categories of bare life and the state of exception, in which law suspends itself and leaves life exposed to power, have been used to analyze state violence in Latin America, for instance in the Colombian armed conflict (Agamben, 1998; López Herrera, 2018). The sterilizations were carried out through ordinary legal instruments instead: a law, a ministerial resolution, protocols and forms. Because the program worked through ordinary law, its instruments remained in the official record after it ended. Foucault’s account of law operating as a norm fits this case better than the paradigm of exception, and the category proposed here focuses on that ordinary operation of legal form.

These frameworks explain how power governs life, how it distributes exposure to death and how it regulates reproduction through laws, programs and rights. The question they leave open is narrower. They do not explain how legal form confers legitimacy on an intervention while it is carried out, or why official bodies can still dispute its legal characterization decades after it has ended. That is a question about law as a source of legitimacy, and the Peruvian case raises it directly.

## 3 Law as symbolic power in an internationalized juridical field

For Bourdieu, the question of who may say what the law is defines a field. In his words, “the juridical field is the site of a competition for monopoly of the right to determine the law” (Bourdieu, 1987, p. 817). García Villegas (2014) describes it as a field of relations among actors who hold different positions and struggle over the symbolic power of legal texts; lawyers, legislators, judges and law professors are chief among them. That struggle has political weight because law classifies, separating the legal from the illegal and the just from the unjust, and actors in different positions compete to say what the law is (García Villegas, 2014). In the Peruvian case, the object of this competition is the legal characterization of the facts of 1996–2000, that is, which official account of those facts carries legal force.

Bourdieu also explains how legal form legitimizes what it authorizes. Juridical language, he observes, bears the marks of a rhetoric of impersonality and neutrality. Its passive and impersonal constructions produce a neutralization effect, making decisions appear to come from no one in particular, and its general formulations produce a universalization effect, making them appear valid for everyone. He counts these effects among the most powerful mechanisms for imposing legitimacy on a social order (Bourdieu, 1987). He also describes how even the lowest-ranking agent, down to the police officer or the prison guard, is tied to the legal theorist by “a chain of legitimation that removes his acts from the category of arbitrary violence” (Bourdieu, 1987, p. 824). Legal decisions thus carry “the symbolic effectiveness possessed by any action which, assuming one ignores its arbitrariness, is recognized as legitimate” (Bourdieu, 1987, p. 828). Applied to a health program, this account suggests that a health worker who performs a sterilization under a ministerial resolution appears to be exercising a recognized authority, not committing an arbitrary act. The neutralization Bourdieu describes is linguistic, whereas the category proposed in Section 4 adds a technical neutralization, which is administrative because it works through targets, quotas and statistics.

García Villegas develops this point for Latin America. In an earlier article he describes one approach to the symbolic dimension of law, in which law is the authorized language through which the State produces and reproduces its legitimacy, because coercion needs a justification and law supplies it (García-Villegas, 2003). He later defines symbolic efficacy as the use of what legal language evokes in order “to achieve political ends different from those set out in the norm itself” (García Villegas, 2014, p. 99; our translation). Norms, in his account, are not always enacted in order to be applied; some are created only in order to be enacted, and some for both purposes (García Villegas, 2014). A program whose recitals invoked the unrestricted exercise of the right to reproductive health while it pursued population goals fits this description (Inter-American Court, 2025, para. 69).

The same efficacy can work against those who first relied on it. For García Villegas, law is at once a power that regulates, imposes and demands obedience and a set of rights that citizens can invoke against that same power (García Villegas, 2014). Readings that stress domination and readings that stress emancipation agree, in his account, that in practice law “can serve both” (García Villegas, 2014, p. 109; our translation). The categories later used to contest the Peruvian program, such as serious human rights violation and reproductive violence, came from the same juridical field that had authorized it. The juridical field that legitimized the program thus also supplied the terms in which that legitimacy could later be withdrawn.

If the same field both authorizes an intervention and later contests it, it matters which bodies belong to that field in the present case. For this analysis, a body belongs to the field when its acts have legal effect in fixing or disputing the characterization of the facts. The criterion follows Bourdieu’s definition of a field by what is at stake in it rather than by a list of institutions. Dezalay and Madsen (2012) add that a field has no fixed boundaries: it has a center where its effects are strongest and outskirts where they weaken, and transnational fields of law develop in interdependence with national ones. Bourdieu himself studied law as part of the formation of the state, of its administration and of the bureaucratic field (Kretschmann, 2025).

On this criterion, the four kinds of bodies that intervened in the case (the executive and its administrative authorities, Congress, prosecutors and domestic courts, and international bodies) occupy unequal positions in one internationalized field. Prosecutors and courts are at its center. The executive and Congress hold their main positions in the bureaucratic and political fields. They enter the juridical field when they issue acts that fix or dispute legal categories, as the ministerial resolution of 1996, the congressional subcommittee’s report of 2002, the decree of 2015 and Law 32107 did (Inter-American Court, 2025, paras. 69, 88, 150; Law 32107, 2024, art. 5). The Inter-American Commission and Court and the CEDAW Committee form the transnational part of the field, which interacts with the domestic part through what Dezalay and Madsen (2012) call double games: victims took their cases to international bodies to change the domestic characterization, and the State defended its measures before them (CEDAW Committee, 2024, para. 4.8). These positions carry unequal resources. The Inter-American Court issues binding judgments, whereas the CEDAW Committee issues Views and recommendations, and even within the domestic center of the field the two highest courts read Law 32107 in opposite ways in 2025 (Constitutional Court of Peru, 2025, legal ground 210; Supreme Court of Justice of Peru, 2025).

Two consequences follow for the argument. The legitimacy of the program at the time of the act was a product of the symbolic efficacy of legal form, which made an intervention in reproductive matters appear to be the regular exercise of official duties. Its later recharacterization is a product of the competition among unequal positions in the same field, which is why the characterizations differed and why, as of September 2026, the dispute remained open.

## 4 Defining the juridical laundering of biopower

We define the juridical laundering of biopower as a two-phase operation of legal form. By legal form we mean the set of legally valid instruments through which a policy is authorized and recorded: statutes, regulations, program documents, protocols and forms. In the first phase, those instruments present a state intervention in the life, health or reproduction of a population as a lawful and technical policy and, when the intervention is framed as voluntary, as one to which the people affected consented. In the second phase, which unfolds over years or decades, official bodies of the same juridical field assign successive and incompatible legal characterizations to that intervention, and the characterization that prevails at any given moment determines whether responsibility can be attributed. The operation therefore acts on the same object as biopower: the life of a population administered by the state. The first phase coincides with the intervention itself, and the second begins with the first official characterization of the facts.

Existing concepts capture parts of this operation, but none of them connects the legitimacy that law gives an intervention while it is under way with the way official bodies later classify the same facts. Reproductive governance identifies the laws, incentives and forms of coercion through which states and other actors seek to control reproduction (Morgan and Roberts, 2012), but it describes those instruments rather than the legitimacy that legal form confers on them. Work on symbolic power and symbolic efficacy explains how law legitimizes coercion, since law is “the authorized language of the State through which its legitimacy is produced and reproduced” (García-Villegas, 2003, p. 170; see also Bourdieu, 1987). That literature, however, does not examine how the same field treats, years later, the policies it once legitimized. Legal violence captures “the normalized but cumulatively injurious effects of the law” (Menjívar and Abrego, 2012, p. 1380), but in that concept law is the source of harm rather than the medium that first authorizes an intervention and later changes its legal characterization.

Three further bodies of work describe uses of law that differ from the one at issue here. Lawfare designates the strategic use of law as a weapon in conflict (Jones, 2016), and it presupposes an actor with a purpose, whereas the operation described here has no single author. Biolegality designates the way legal and biological categories validate each other and, in one strand of political philosophy, the sovereign’s violence when it is justified in the name of life (van Wichelen and de Leeuw, 2022). Studies of accountability for past state violence deal mainly with crimes such as disappearance and torture, committed outside the law of the time and later shielded by amnesties (Laplante, 2009; Lutz and Sikkink, 2001; Roht-Arriaza, 2015). The sterilizations, by contrast, were carried out through the legal instruments of a public program. The category proposed here brings together what these concepts treat separately: an intervention in the life of a population, the legitimacy that legal form gives it, and the later dispute over its legal characterization.

The term laundering is an analogy: it names the conversion of an intervention that would be contested if openly described into a lawful and technical policy to which those affected consented. The analogy stops there, because the offense of money laundering requires assets derived from a prior offense and an agent who conceals their origin (Legislative Decree 1106, 2012; Loli Ausejo, 2024). The operation described here has no single author, and at the time of the intervention no offense had been recognized as such.

The category applies when three conditions hold. First, a state intervenes in the life, health or reproduction of a population. Second, the intervention is authorized and recorded through legally valid instruments. Third, official bodies later assign it more than one legal characterization. Violence carried out outside legal form, such as clandestine detention, falls outside the category, and so do isolated abuses unconnected to a policy, including sterilizations performed without consent outside any program. Where the third condition is absent, only the first phase can be observed.

The Peruvian program is the case we use to specify and illustrate the category rather than to establish how broadly it applies. The three conditions also hold in the Czechoslovak sterilization program, which from 1972 targeted mainly Romani women. A 1971 directive set strict consent requirements, including review by a commission, and guidelines adopted in 1973 allowed a one-off payment for “certain medical treatments in the interest of a healthy population” (Rowlands et al., 2025, pp. 9–11). The performance of social workers was measured by the number of women from whom they obtained consent, and cases were later taken to the European Court of Human Rights (Rowlands et al., 2025). One case cannot show how often the operation occurs, or whether its second phase takes the same course elsewhere. Our evidence concerns reproductive policy, and whether the operation extends to other interventions in the life of populations remains to be examined.

The operation unfolds in four moments, which we distinguish by the instrument each uses, the question each settles and the effect each produces (Table 2). Legal inscription makes the intervention lawful: a statute, a regulation or a program document authorizes it and states its purpose. Technical neutralization turns the policy into a matter of administration, since targets, protocols, incentives and statistics convert a decision about a population into a question of coverage and performance. Consent as sign individualizes the intervention, because a signed form records it as the decision of the person who undergoes it. Deferred recharacterization belongs to the second phase, where prosecutors, legislators, courts and international bodies assign the facts a legal category that governs whether responsibility can be attributed. The first three moments operate while the intervention takes place; the fourth operates afterwards, whenever an official body has to decide what the facts were in legal terms.

**Table 2:** The four moments of the juridical laundering of biopower.

Moment	Phase	Instrument and author	Question it settles	Effect	Illustration in the Peruvian case
Legal inscription	First	Statutes, regulations and program documents, issued by the legislature and the executive	Is the intervention lawful?	Authorization: the intervention acquires legal validity and a lawful stated purpose	The ministerial resolution presented the program as an instrument guaranteeing the right to reproductive health and as the means to “adequate population growth in accordance with the country’s development” (Inter-American Court, 2025, para. 69; our translation)
Technical neutralization	First	Targets, quotas, protocols, incentives and statistics, produced by the health administration	Is the intervention a political decision or a matter of health management?	Depoliticization: decisions appear as coverage and performance	The target that 100% of women receiving institutional care for childbirth or abortion leave having started a contraceptive method (Inter-American Court, 2025, para. 70); quotas described as demand-based targets (para. 54)
Consent as sign	First	Counseling protocol and individual consent form, completed by health personnel and patient	Whose decision was the intervention?	Individualization: each intervention is recorded as the decision of the person who underwent it	A procedures manual requiring counseling and a signed form (Inter-American Court, 2025, paras. 72–73); signatures obtained after surgery or under pressure (para. 67)
Deferred recharacterization	Second	Prosecutorial, legislative, judicial and international decisions, issued by bodies of the juridical field	What is the intervention, legally, and who is responsible for it?	Responsibility attributed or not: the characterization that prevails determines whether responsibility can be attributed	34 official acts between 1997 and 2026 (Section 6)

The moments are separable, as the case itself shows. By March 1998 the Ministry of Health had overhauled the consent procedure, adding a second counseling session, a 72-hour interval and a new form. It told the Ombudsman’s Office, however, that the modification of its targets was still pending, and it reworded only one service target (Inter-American Court, 2025, paras. 59, 71, 73). Consent as sign could thus be reformed while technical neutralization remained largely in place. That moment also presupposes an intervention presented as voluntary; where a law makes sterilization compulsory, it has no place.

The connection between the phases is documentary, because the instruments produced in the first phase are reused in the second. The dismissal of January 2014 relied on the law that authorized surgical contraception, on the program’s public-health purpose and on the manual that governed consent (prosecutorial decision of 22 January 2014, quoted in Novoa Curich, 2014). The dismissal of December 2016 relied on a signed consent form (Inter-American Court, 2025, para. 93). Before the CEDAW Committee, the State relied on the existence of measures guaranteeing free, prior, full and informed consent (CEDAW Committee, 2024, para. 4.8).

The category can be refuted by observation. It would fail if the instruments of the first phase presented the intervention openly as coercive or punitive, because legal form would then confer no appearance of lawfulness, technique or consent. It would fail if the same facts kept a single legal characterization across bodies and over time, because there would then be no second phase to describe. It would fail if later decisions reasoned without any reference to the instruments of the first phase, because the two phases would then be unconnected. The category does not predict impunity. Whether the second phase ends in the attribution of responsibility or in its absence is an empirical question, and the Peruvian case answers it in both directions.

## 5 Legitimation of the program, 1995–2000

Between 1996 and 2000 the sterilizations were carried out as a public policy authorized by a law amended in 1995. The family planning program had been reformed after the 1994 Cairo conference on population and the 1995 Beijing conference on women, and its guiding documents largely reflected the language agreed upon at Cairo, including reproductive rights and gender equity (Ewig, 2006). Three kinds of instruments gave the program its legitimacy while it operated: a law and a ministerial resolution, a system of numerical sterilization targets, and a procedure for consent. Each corresponds to one of the three moments of the first phase (Table 2).

Legal inscription began with the law. In 1995, through Law 26530, Congress amended the National Population Policy Act to authorize voluntary surgical contraception, known in Spanish as *anticoncepción quirúrgica voluntaria* (CEDAW Committee, 2024, para. 2.1; Novoa Curich, 2014). On 6 February 1996, Ministerial Resolution 071-96-SA/DM approved the National Reproductive Health and Family Planning Program for 1996–2000 (Inter-American Court, 2025, para. 69). Its recitals presented the program as “a technical and regulatory instrument that guarantees the unrestricted exercise of the right to reproductive health.” That instrument, in turn, was the means of achieving the population policy’s aim of “adequate population growth in accordance with the country’s development” (Inter-American Court, 2025, para. 69; our translation). Tamayo (1999) found that a demographic target was among the program’s general goals. The very name of the procedure, voluntary surgical contraception, also built consent into the legal category.

The same instruments used two vocabularies at once, one of rights and one of demographic goals. Ewig (2006, p. 635) argues that national political actors “appropriated national and global feminist discourses to legitimize their actions.” In a later study she concludes that, beneath its rights-oriented rhetoric, the program’s primary goals were economic growth and poverty reduction, and that the president and the highest levels of his administration covertly favored population control over reproductive rights (Ewig, 2010). For Silva Santisteban (2021), the sterilizations were a policy of population reduction through birth control, whose objective was economic rather than the expansion of women’s rights. Legal inscription thus gave the program a lawful purpose in both vocabularies, and the language of rights made the demographic aim harder to see.

Technical neutralization turned the program into a matter of coverage. One service target required that every woman receiving institutional care for childbirth or abortion be discharged having started a safe contraceptive method after individual counseling (Inter-American Court, 2025, para. 70). Numerical targets for surgical contraception rose every year under the supervision of the central authority, and quotas for recruiting users were assigned to facilities and health workers (Tamayo, 1999). The congressional investigative subcommittee later found numerical targets, incentives and quotas for recruiting users nationwide, and the Ombudsman’s Office found centers that required a set number of sterilizations each month (Inter-American Court, 2025, paras. 60, 67). According to a report of the Peruvian Medical Association, officials said that the targets came from the highest political level and had to be met (Inter-American Court, 2025, para. 57). The State used the number of women sterilized as an indicator of success in reducing poverty, and the quota system helped produce the 217,446 sterilizations of women recorded for 1996–1998 (Ewig, 2006). Facilities that met their quotas received equipment and professionals received bonuses, while those who did not faced sanctions and the denial of promotion (Boesten, 2007; Carranza Ko, 2020).

The quotas appear in the program’s own paperwork. A document of the Huancavelica health sub-region assigned two or three patients a month to each staff member and stated that no payment was due for recruiting them, because the task was mandatory (Tamayo, 1999). On 11 October 1997, the director of the Huancabamba health center told a worker in a memorandum that he or she “must recruit two patients for voluntary surgical contraception this October” (Tamayo, 1999, p. 56; our translation). The memorandum described the task as mandatory and warned that otherwise a report would be filed to terminate the worker’s contract. The Ministry of Health, for its part, told the Ombudsman’s Office that its targets rested on estimates of unmet demand and implied neither quotas nor sanctions (Inter-American Court, 2025, para. 54). The official figures also differed: the government reported 37,000 surgical contraceptions for 1996, while national statistics recorded 81,761 tubal ligations and 6,313 vasectomies in the public sector that year (Tamayo, 1999). The administrative vocabulary itself performed the neutralization: quotas became demand-based targets, and the number of operations became a measure of coverage.

Consent as sign rested on a procedure and a form. The 1996 manual for surgical contraception made counseling a prerequisite for the operation and instructed counselors “not to overwhelm users by offering them information they do not need” (Inter-American Court, 2025, para. 72; our translation). In 1998, following recommendations of the Ombudsman’s Office, the Ministry added a second counseling session, a minimum interval of 72 h before surgery and a new consent form (Inter-American Court, 2025, para. 73). The program’s public messaging used the same register, as in a mural that read “Only you can decide how many children to have” (Ewig, 2006, p. 632, fig. 1). Norms of this kind do not by themselves secure autonomy: Gianella (2013) observes that in Peru, despite specific norms and laws, the exercise of patient autonomy is uncommon and paternalism prevails in medical decisions.

In practice, the signature recorded, for many women, a decision they had not made. Of the 56 women interviewed by the Ombudsman’s Office, 35 said they had signed an authorization; of these, 2 had signed after the operation, and 6 had signed without knowing exactly what the document was or under pressure. One third considered that the method had been imposed on them (Inter-American Court, 2025, para. 67). More than 65% of the women who were sterilized could not read or write or had not completed primary school, and many spoke Quechua (Málaga, 2013). Tamayo (1999) documented sterilizations performed despite the woman’s withdrawal of consent, under intimidation or confinement, and through deception about the irreversibility of the operation. Of the women who brought their cases to the CEDAW Committee, one describes being made to sign a document “for medicines,” and another was asked to sign one in order to receive food for her children (CEDAW Committee, 2024, paras. 2.10–2.11). In these conditions the form certified a decision without showing how it had been obtained.

The program reached some women more than others. The congressional investigation found that it gave priority to Indigenous women from very poor rural, peri-urban, Andean and Amazonian areas (Inter-American Court, 2025, para. 52). The CEDAW Committee observed that at least 314,000 women, 93% of those affected, and 24,000 men were sterilized under the program, most of them Indigenous, unable to read or write, or from poor or rural areas (CEDAW Committee, 2024, para. 7.7). Tamayo (1999) had already recorded discrimination by gender, poverty and ethno-linguistic origin. The Inter-American Court concluded that the policy was directed mainly at women made vulnerable by their ethnicity and socioeconomic status (Inter-American Court, 2025, para. 152). Human rights scholarship has argued more generally that forced sterilization is at its core a form of discrimination, which courts deciding such cases have rarely named (Patel, 2017). Scholars disagree on how to characterize this selectivity. Carranza Ko (2020) and Silva Santisteban (2021) argue that the sterilizations amount to genocide, while De La Cruz Huamán (2018) reports that most research does not characterize them as ethnic genocide but recognizes their socioeconomic and ethnic discrimination. Our argument does not depend on settling that question, which is itself part of the later dispute over the characterization of the facts.

At the time of the intervention, the program appeared lawful, technical and consented to. A law and a ministerial resolution gave it a lawful purpose, expressed in the language of rights as well as in that of demographic goals. Targets and quotas presented its operation as a matter of coverage, and signed forms recorded each intervention as the decision of the woman who underwent it. These documents did not disappear when the program ended; they remained in official files, where later decisions could draw on them. The selectivity of the program, by contrast, was not visible in instruments that spoke of rights, coverage and consent.

## 6 Legal characterizations of the program, 1997–2026

A legal characterization, as defined in Section 1, places the facts in a legal category, and that category affects whether, and against whom, responsibility can be attributed. Table 3 lists 34 official acts that made or disputed such characterizations of the sterilizations performed under the program between 1997 and 2026. They come from prosecutors, Congress, the executive, the Ombudsman’s Office, the Truth and Reconciliation Commission, domestic courts, the National Board of Justice and international bodies. Prosecutors issued 9 of these acts and domestic courts 10, and each row of the table records a single act. Domestic courts have issued judgments in individual criminal cases and on reparations, but none has decided on the responsibility of those who designed and directed the program.

**Table 3:** Official acts that characterized the facts of the sterilization program, 1997–2026.

Year	Body	Act	Characterization of the facts	Consequence for responsibility	Source
1997	Provincial prosecutor, Piura	Dismissal (Ramos Durand)	Fortuitous event; out-of-court settlement	None	Inter-American Court (2025, para. 86)
1998	Ombudsman’s Office	Report 7	Coverage targets only for women; campaigns favoring definitive methods	Recommendations	Inter-American Court (2025, paras. 58, 66)
1998	Ministry of Health	Letter to the Ombudsman’s Office	Targets based on demand, not quotas	Denial	Inter-American Court (2025, para. 54)
1999	Ombudsman’s Office	Report 27	Monthly sterilization requirements in health centers; defective consent documents	Recommendations	Inter-American Court (2025, paras. 67–68)
2000	Inter-American Commission	Second Report on the Situation of Human Rights in Peru	The program gave rise to forced sterilizations	Non-binding finding	Inter-American Court (2025, para. 62)
2001	State and Inter-American Commission	Preliminary friendly settlement agreement (Mestanza)	International responsibility of the State admitted	Commitment to a final settlement	Inter-American Commission (2003, paras. 7–8)
2002	Congressional subcommittee	Final Report	Indications of crimes against liberty and against life and health, criminal association, genocide	Constitutional complaint	Inter-American Court (2025, para. 88)
2003	Congress	Plenary vote	Complaint shelved	None	Inter-American Court (2025, para. 88)
2003	Truth and Reconciliation Commission	Final Report	Sterilizations excluded	None	Getgen (2009)
2003	State and Inter-American Commission	Friendly settlement (Mestanza)	Violations of the American Convention and the Belém do Pará Convention	State responsibility acknowledged	Inter-American Commission (2003, paras. 7, 14)
c. 2003	Court, Piura	Judgment (Vigo case)	Individual liability of the physician	Judgment against the physician	Páez (2009)
2004	Prosecutor	Criminal complaint based on the second constitutional complaint shelved	Not stated	None	Inter-American Court (2025, para. 90)
2009	Provincial prosecutor specialized in human rights	Dismissal of 2,074 complaints	Ordinary offenses barred by limitation	None; confirmed on appeal	Inter-American Court (2025, paras. 64, 89)
2011	Attorney General’s Office	Reopening	Duty to investigate under the friendly settlement	Investigation resumed; extended to Fujimori in 2013	Inter-American Court (2025, para. 91)
2014	Supraprovincial prosecutor	Dismissal	Lawful public-health program without criminal intent	None	Inter-American Court (2025, para. 92) and Novoa Curich (2014)
2015	Senior prosecutor	Annulment of the dismissal	Indirect perpetration and crimes against humanity to be examined	Investigation resumed	Inter-American Court (2025, para. 92)
2015	Executive	Supreme Decree 006-2015-JUS	Forced sterilizations; victims of national interest	Registry of victims; no attribution	Inter-American Court (2025, para. 150)
2016	Supraprovincial prosecutor	Charges and dismissal (July)	Physicians charged (seven women); no charges against Fujimori and the main suspects	Partial	Inter-American Court (2025, para. 93)
2016	Supraprovincial prosecutor	Confirmation of dismissal (December)	Signed consent form: no sign that Celia Ramos’s decision was vitiated	None	Inter-American Court (2025, para. 93)
2017	Executive (President)	Supreme Resolution 281-2017-JUS	None: humanitarian pardon and right of grace covering convictions and pending proceedings	Proceedings against Fujimori at risk of extinction	Inter-American Court (2025, para. 94)
2018	Senior prosecutor	Order to charge	Serious bodily injury followed by death; indirect perpetration	Charges against Fujimori and others	Inter-American Court (2025, para. 95)
2021	Criminal court	Order opening criminal proceedings	As in the 2018 charges	Proceedings opened	Inter-American Court (2025, para. 96)
2022	National Superior Court (criminal chamber)	Judgment	Serious bodily injury constituting a serious human rights violation	Acquittal	Supreme Court of Justice of Peru (2025, preamble)
2022	Constitutional court, Lima	*Amparo* judgment	Constitutional right to reparation	Reparations policy ordered	CEDAW Committee (2024, para. 2.6)
2023	Constitutional chamber, Lima Superior Court	*Amparo* appeal judgment	Constitutional right to reparation	Order confirmed	CEDAW Committee (2024, para. 2.6) and Inter-American Court (2025, para. 246)
2023	Supreme Court, Constitutional and Social Law Chamber	*Amparo*	Inadequate reasoning (according to the State)	Order opening proceedings annulled	Inter-American Court (2025, para. 97) and CEDAW Committee (2024, para. 6.1)
2024	Criminal chamber	Judgment (Mestanza case)	Exposing a dependent person to danger, in a context of human rights violations	One physician convicted (suspended sentence); others acquitted; appeal	DEMUS et al. (2024)
2024	National Superior Court (criminal chamber)	Appeal decision	Annulment of the 2021 order confirmed	Proceedings of 2021–2023 voided	El Comercio (2024)
2024	Congress	Law 32107	Facts before July 2002 cannot be crimes against humanity and are subject to limitation	Prosecution restricted	Law 32107 (2024, arts. 4–5)
2024	CEDAW Committee	Views	Gender-based violence; intersectional discrimination; recalled that widespread or systematic forced sterilization is a crime against humanity, without concluding so in the case	State responsibility	CEDAW Committee (2024, paras. 8.6, 8.9)
2025	Constitutional Court	Judgment 190/2025	Law 32107 upheld; limitation suspended until January 2002	Limitation rules disputed	Constitutional Court of Peru (2025, operative paragraphs 1–2, legal ground 210)
2025	Inter-American Court	Judgment	State policy; institutional and reproductive violence; serious human rights violation	State responsibility; limitation inapplicable in the investigation	Inter-American Court (2025, paras. 152–153, 176, 225)
2025	Supreme Court, Transitory Criminal Chamber	Appeal for annulment 1684-2022	Serious human rights violations not subject to ordinary limitation	Acquittal annulled; retrial ordered; Law 32107 inapplicable	Supreme Court of Justice of Peru (2025, summary and decision I–III)
2026	National Board of Justice	Preliminary disciplinary inquiry (reported)	Refusal to apply Law 32107 examined as possible misconduct	Pending	IDEHPUCP (2026) and La República (2026)

The first characterizations treated the facts as a fortuitous event, as rights violations in the program’s implementation, as possible crimes, or not at all. In December 1997 a provincial prosecutor in Piura dismissed the complaint over Celia Ramos’s death as a fortuitous event, an accident for which no one was legally responsible, and noted that her husband had withdrawn the complaint under an out-of-court settlement. The Ombudsman’s Office considered the dismissal unlawful and found a causal link between the sterilization and her death (Inter-American Court, 2025, paras. 86–87). In its reports of 1998 and 1999 it found coverage targets set only for women, campaigns that favored definitive methods and health centers that required a set number of sterilizations each month (Inter-American Court, 2025, paras. 66–67). The Ministry of Health replied in 1998 that its targets rested on estimated demand and did not mean quotas (Inter-American Court, 2025, para. 54).

The characterizations of the following years came from Congress, prosecutors and the Truth and Reconciliation Commission. In its 2002 final report, a congressional subcommittee found indications of crimes against individual liberty, crimes against life, body and health, criminal association and genocide. It filed a constitutional complaint against Fujimori and his health ministers, which the full Congress shelved in March 2003 (Inter-American Court, 2025, para. 88). A second constitutional complaint, filed in August 2003, was declared admissible. The congresswoman who had filed it then lodged a criminal complaint with the same content, which the prosecutor’s office shelved in July 2004 (Inter-American Court, 2025, para. 90). The Truth and Reconciliation Commission, whose Final Report appeared in August 2003, left the sterilizations out of its investigation and report. Getgen (2009) documents this exclusion and argues that it erased State responsibility for the sterilizations and reduced the prospects of justice and reparations for the women affected.

In 2009 the facts were treated as ordinary offenses barred by the statute of limitations. On 26 May 2009 a provincial prosecutor specializing in human rights dismissed the consolidated investigation, which by then comprised 2,074 complaints, by applying the statute of limitations. Appeals against that decision were rejected in December 2009 (Inter-American Court, 2025, paras. 64, 89). The Attorney General’s Office reopened the investigation in October 2011. It cited the concern expressed by the Inter-American Commission and the State’s undertaking, in the Mestanza friendly settlement, to investigate, identify and punish those responsible (Inter-American Court, 2025, para. 91 and fn. 139). According to the authors of a later communication to the CEDAW Committee, the Attorney General’s Office also argued that the 2009 dismissal was not *res judicata*, that is, not a final decision barring new proceedings. In its view, the offenses had not yet been investigated as crimes against humanity (CEDAW Committee, 2024, para. 2.4).

When the investigation reached the officials who had designed and directed the program, prosecutors twice declined to charge them on grounds drawn from the program’s own instruments. In November 2013 the investigation was extended to Fujimori as an indirect perpetrator (*autoría mediata*). On 22 January 2014 the prosecutor declined to charge him and the other main suspects and dismissed the case for the second time (Inter-American Court, 2025, paras. 91–92; Novoa Curich, 2014). The reasoning rested on three premises. First, surgical contraception had been introduced by Law 26530, which Congress passed under the regular legislative procedure. Second, the program pursued a public-health goal, the reduction of very high maternal and infant mortality. Third, its procedures manual was designed to secure counseling, free choice and prior information before consent. From these premises the prosecutor inferred that the program was “devoid of *dolo*, that is, of criminal intent” (prosecutorial decision of 22 January 2014, pp. 42–43, quoted in Novoa Curich, 2014; our translation).

In April 2015 a senior prosecutor annulled the dismissal and ordered inquiries into indirect perpetration and into a possible characterization as crimes against humanity (Inter-American Court, 2025, para. 92). In July 2016 prosecutors again declined to charge Fujimori and the main suspects, brought charges against some physicians in the cases of seven women, and dismissed the case of Celia Ramos. In December 2016 they confirmed that dismissal because she had signed a consent form, which in their view left no objective indication that her consent had been obtained improperly (Inter-American Court, 2025, para. 93).

Between 2017 and 2024 criminal proceedings against Fujimori and senior officials were first put at risk, then opened and then undone. In December 2017 the President granted Fujimori, on humanitarian grounds, a pardon for his convictions and a right of grace (*derecho de gracia*), a form of clemency that ends pending criminal proceedings (Inter-American Court, 2025, para. 94). In April 2018, following an appeal by the representatives of Celia Ramos, a senior prosecutor ordered that Fujimori and others be charged as indirect perpetrators of serious bodily injury followed by death in the cases of five women, including Celia Ramos. The same order required an investigation of serious bodily injury concerning 2,074 women who had survived their operations (Inter-American Court, 2025, para. 95). On 11 December 2021 a criminal court issued an order opening criminal proceedings (*auto de apertura de instrucción*) against Fujimori and those principally responsible for implementing the program (Inter-American Court, 2025, para. 96).

On 25 August 2023 the Constitutional and Social Law Chamber of the Supreme Court annulled that order in a constitutional protection action (*amparo*) brought by one of the accused (Inter-American Court, 2025, para. 97). According to the State, the order was annulled for inadequate reasoning, and the investigation itself was not voided (CEDAW Committee, 2024, para. 6.1). In November 2023 the trial court returned the file to the prosecutors, and in March 2024 it brought the case under the new Code of Criminal Procedure (Inter-American Court, 2025, para. 97 and fn. 149). In August 2024 a criminal chamber of the National Superior Court rejected the prosecution’s appeal and upheld the annulment, which voided the proceedings of 2021–2023 (El Comercio, 2024). Fujimori died in September 2024 without having been tried for the sterilizations, and his death ended the criminal proceedings against him (El Comercio, 2024; Infobae, 2024; Criminal Code of Peru, 1991, art. 78). The case against the former ministers remained with the prosecutors when the Inter-American Court delivered its judgment (Inter-American Court, 2025, para. 98).

The domestic criminal judgments documented in the sources examined here concern physicians, not the officials who designed the program. Victoria Vigo, sterilized without her consent during a cesarean section in a public hospital in Piura in 1996, brought the physician to trial and obtained a judgment against him about seven years later. In 2009 the press described it as the only judgment on the sterilizations (Páez, 2009). In July 2024 a criminal chamber convicted the physician who had refused to treat Mamérita Mestanza when she returned with complications; the offense was exposing a dependent person to danger in a context of human rights violations. He received a suspended four-year sentence, the other defendants were acquitted, and the victims’ representatives appealed (DEMUS et al., 2024).

In August 2022 a criminal chamber of the National Superior Court acquitted physicians charged with serious bodily injury constituting a serious human rights violation (Supreme Court of Justice of Peru, 2025, preamble). The charges concerned sterilizations performed without consent on several women, including at a hospital in Chincheros, Apurímac, in 1998 and 2000, and at a health center in La Unión, Huánuco, in 1997. One of the women had hearing and speech disabilities and a psychiatric condition (Supreme Court of Justice of Peru, 2025). On 23 December 2025 the Supreme Court annulled the acquittal and ordered a new trial before a different panel. It found that the chamber had analyzed neither the acts attributed to each defendant nor the vulnerable position of the women (Supreme Court of Justice of Peru, 2025, summary and decision II–III). No information on the new trial was available as of September 2026.

Outside criminal law, the executive and the constitutional courts recognized the women as victims with a right to reparation, without attributing criminal responsibility. In November 2015 a supreme decree declared the priority care of victims of forced sterilizations carried out between 1995 and 2001 a matter of national interest and created the Registry of Victims of Forced Sterilizations. The Inter-American Court later observed that, by doing so, the State had itself characterized these acts as forced sterilizations (Inter-American Court, 2025, para. 150). In November 2022 a constitutional court in Lima recognized a constitutional right to reparation and ordered the Ministry of Justice to adopt a reparations policy for the registered women, an order confirmed on appeal in July 2023 (CEDAW Committee, 2024, para. 2.6; Inter-American Court, 2025, para. 246). The policy had not been implemented by 2024 (CEDAW Committee, 2024, para. 8.6). Before the CEDAW Committee, the State argued that registration in the registry did not imply any recognition of responsibility, which only the judiciary could establish (CEDAW Committee, 2024, para. 6.2). Bills to extend the national reparations law to these women have been introduced since 2008 (Ruiz Alvarado, 2024).

International bodies characterized the facts as violations for which the State is responsible. In 2000 the Inter-American Commission found that the program had given rise to forced sterilizations (Inter-American Court, 2025, para. 62). In the Mestanza case, Peru acknowledged its international responsibility, first in a preliminary agreement of March 2001 and then in a friendly settlement signed in August 2003 (Inter-American Commission, 2003, paras. 7–8). It acknowledged violations of the rights to life, personal integrity and equal protection under the American Convention and of the Belém do Pará Convention. It also undertook to investigate and punish those responsible and to reform its reproductive health legislation and policies (Inter-American Commission, 2003, para. 14). In 2024 the CEDAW Committee held that the facts constituted gender-based violence against women and intersectional discrimination based on sex, gender, rural origin and socioeconomic status (CEDAW Committee, 2024, para. 8.6). It recalled that forced sterilization may amount to torture or cruel, inhuman or degrading treatment, and observed that the repeated return, shelving and closure of the proceedings had revictimized the authors (CEDAW Committee, 2024, paras. 8.3, 8.7).

In 2025 the Inter-American Court concluded that the program was a State policy aimed at promoting mass sterilization as a contraceptive method, pursued through threats and coercion against women made vulnerable by their ethnicity and socioeconomic status. That policy was organized and directed from the highest levels of the State (Inter-American Court, 2025, paras. 152–153). The Court characterized the sterilization of Celia Ramos as institutional violence against women and as reproductive violence, and held Peru responsible for violating, among others, the rights to life, personal integrity and health (Inter-American Court, 2025, paras. 153, 176; operative paragraph 3). It ordered a public act of acknowledgment, the registration as victims of the women who died after being sterilized, and the design of the reparations policy that a domestic constitutional court had already ordered (Inter-American Court, 2025, operative paragraphs 10, 17–18). The judgment, dated 25 November 2025, was notified and made public on 5 March 2026 (Inter-American Court, 2026).

Since 2024 the dispute has centered on the passage of time: whether statutory limitation bars the prosecution of facts that occurred between 1996 and 2000. Law 32107, published in August 2024, provides that no fact prior to 1 July 2002 may be characterized as a crime against humanity. It also provides that offenses committed before the relevant treaties entered into force for Peru are subject to national limitation periods (Law 32107, 2024, arts. 4–5). The CEDAW Committee noted that forced sterilization, when widespread or systematic, constitutes a crime against humanity under the Rome Statute, but stated that reaching such a conclusion in the case was outside its purview (CEDAW Committee, 2024, para. 8.9). On 18 November 2025 three justices of the Constitutional Court voted to strike the law down, short of the five votes required. The other four upheld it, on the reading that limitation periods under its Article 4 did not run until January 2002, a date tied to the annulment of military-court judgments. No sterilization case was before the Court (Constitutional Court of Peru, 2025, operative paragraphs 1–2, legal ground 210).

A week later the Inter-American Court held that, because the sterilization of Celia Ramos was a serious human rights violation, “no amnesty, statute-of-limitation or exclusion-of-responsibility provisions may be applied” in its investigation. It made this binding in an operative paragraph adopted unanimously (Inter-American Court, 2025, para. 225, operative paragraph 8; our translation). The majority did not address whether the facts were crimes against humanity, and Judge Hernández López, who presided over the Court when the judgment was adopted, argued in a concurring opinion that they should have been so characterized (Inter-American Court, 2025, concurring and partly dissenting opinion of Judge Pérez Manrique, para. 113; concurring opinion of Judge Hernández López, paras. 1, 47). On 23 December 2025 the Supreme Court rejected a defendant’s plea of statutory limitation. It held that facts characterized as serious human rights violations are not subject to the limitation rules of ordinary proceedings and that “Law 32107 is inapplicable” to the case (Supreme Court of Justice of Peru, 2025, p. 32, decision I; our translation). In June 2026, according to press reports, the National Board of Justice opened a preliminary inquiry into the five justices who had issued that decision (IDEHPUCP, 2026; La República, 2026). The two highest domestic courts thus disagree on statutory limitation, while the Supreme Court and the Inter-American Court reach the same result through the category of serious human rights violation.

Across these acts, the same facts have received at least six legal characterizations. They have been treated as a fortuitous event, as ordinary offenses barred by limitation and as the lawful execution of a public-health program. They have also been treated as offenses committed by individual physicians, such as serious bodily injury or exposing a dependent person to danger. Most recently, they have been treated as serious human rights violations for which the State is responsible, including gender-based, institutional and reproductive violence. A sixth characterization, genocide or crimes against humanity, has been claimed by members of Congress, by victims and by one judge, but no court that has decided on the facts has adopted it. Responsibility has been attributed to the State before international bodies and, in two individual cases, to physicians before domestic courts, but not to the officials who designed and directed the program. The former president died without having been tried for the sterilizations, and the country’s two highest courts remain divided on statutory limitation.

## 7 Discussion

In the Peruvian case, the successive recharacterizations of the facts have produced accountability for the State while delaying it for individuals. At the level of the State, Peru acknowledged its responsibility in the friendly settlement of 2003, the CEDAW Committee found in 2024 that it had violated the Convention, and the Inter-American Court declared it responsible in 2025 (Inter-American Commission, 2003, para. 14; CEDAW Committee, 2024, para. 8.8; Inter-American Court, 2025, operative paragraph 3). Domestic constitutional courts, for their part, ordered a reparations policy for registered victims, which the Inter-American Court then ordered Peru to design and begin implementing (CEDAW Committee, 2024, para. 2.6; Inter-American Court, 2025, para. 251, operative paragraph 18). At the level of individuals, the criminal convictions documented in our sources concern physicians: the one who sterilized Victoria Vigo (Páez, 2009) and the one who refused to treat Mamérita Mestanza, convicted in July 2024 and given a suspended sentence that was under appeal (DEMUS et al., 2024). The acquittal of other physicians was annulled in 2025 with an order for a new trial (Supreme Court of Justice of Peru, 2025, decision II–III). No official who designed or directed the program has been brought to trial, and Fujimori died in 2024 without having been tried in this case (Infobae, 2024). We therefore use deferred impunity in a narrow sense: a prolonged failure to hold any individual criminally responsible, which can coexist with the responsibility of the State.

A legal characterization matters because it is an act of naming that carries legal consequences. For Bourdieu (1987), a court’s judgment is an act of naming, and law is the strongest form of the symbolic power to name, because it gives what it names a permanence that few other social acts can confer. Cover (1986, p. 1601) states the practical consequence: “Legal interpretive acts signal and occasion the imposition of violence upon others.” Each category in Table 3 carries its own legal rules. Ordinary offenses become time-barred, and the consolidated complaints were dismissed on that ground in 2009 (Inter-American Court, 2025, paras. 64, 89). For the Inter-American Court and for the Supreme Court, a serious human rights violation is not subject to statutory limitation (Inter-American Court, 2025, para. 225; Supreme Court of Justice of Peru, 2025). Under Law 32107, no fact prior to July 2002 may be characterized as a crime against humanity (Law 32107, 2024, art. 5), and a characterization made by an international court concerns the responsibility of the State, not that of individuals (Inter-American Court, 2025, concurring opinion of Judge Hernández López, para. 48). The choice of category thus determines who can be held responsible and for how long prosecution remains possible.

Bourdieu’s account also suggests why characterizations change over time. For him, the body of law records the balance of power at a given moment, and he notes that the legal status of American labor unions moved from criminal conspiracy to full recognition as their power grew (Bourdieu, 1987). Acts of naming, in his account, succeed when they confirm changes already under way. Lutz and Sikkink (2001) describe one such development: a rapid shift in Latin America toward recognizing the legitimacy of human rights norms, within which prosecutions for past abuses spread. In the Peruvian case, the categories that prevail in the most recent decisions are serious human rights violation and gender-based, institutional and reproductive violence. International bodies adopted them after the victims took their cases there (CEDAW Committee, 2024, para. 8.6; Inter-American Court, 2025, paras. 153, 176, 225). We offer this as an interpretation of the sequence, not as a causal finding.

The effects of the first phase do not end when the intervention ends, as the dismissals of 2014 and 2016 show (Section 6). For Bourdieu, an action acquires symbolic effectiveness when it is recognized as legitimate and its arbitrariness is ignored (Bourdieu, 1987). The bodies that reached a different characterization were those that looked beyond the instruments to what had actually happened: the Inter-American Court applied a stricter standard of consent and found a State policy pursued through threat and coercion (Inter-American Court, 2025, paras. 131, 152). The CEDAW Committee relied on the authors’ coherent and consistent accounts of coercion, pressure and deception and on their inclusion in the victims’ registry (CEDAW Committee, 2024, para. 8.4), and the Supreme Court annulled an acquittal that had disregarded the women’s vulnerability (Supreme Court of Justice of Peru, 2025, summary). This persistence of the first phase’s instruments in the second phase is what we call the long temporality of the symbolic efficacy of law.

A comparison with the Barrios Altos and La Cantuta cases, two sets of killings committed in the same period and under the same government, helps explain why accountability arrived earlier there, even though it later proved reversible. Those killings were the work of the Colina Group, a clandestine military unit acting outside the law of the time, and their perpetrators were shielded afterwards by two amnesty laws passed in 1995 (Burt, 2009). In 2001 the Inter-American Court declared those laws without legal effect, and it later held that this finding applied to every case the laws covered (Laplante, 2009). Fujimori was convicted in 2009, in a context shaped by the transitional government and by key judicial bodies (Burt, 2009). The Peruvian courts characterized the killings as crimes against humanity under international criminal law (Inter-American Court, 2026, para. 5). In those cases the three conditions held. Domestic and inter-American bodies agreed in treating the killings as grave violations that could not be amnestied, and the Inter-American Court’s judgments bound the Peruvian authorities. The Constitutional Court, for its part, had accepted that crimes against humanity are not subject to statutes of limitations (Burt, 2009; Laplante, 2009). The sterilizations, by contrast, received their legal form before and during the intervention, through instruments that were not unlawful in themselves, since surgical contraception is lawful when valid consent is obtained (Inter-American Court, 2025, paras. 116–117, 131). No single decision could deprive those instruments of effect, and each case requires showing that consent was not valid. The sterilizations also lacked the support of transitional institutions, having been left out of the Truth and Reconciliation Commission’s report (Getgen, 2009). The comparison holds period, government and courts constant, but the cases differ in other respects, so it suggests the mechanism rather than establishing it.

These contrasts allow us to state the conditions of each outcome. Recharacterization produces accountability when different official bodies agree on the new legal characterization, a binding decision enforces it, and the rules on statutory limitation allow prosecution. Recharacterization produces deferred impunity when official bodies disagree on the legal characterization, no binding decision enforces any of the competing characterizations, and statutory limitation remains in dispute. The legal form of the first phase makes such agreement harder, because it supplies evidence and arguments to those who resist a new characterization. In the sterilization case, international bodies agreed in 2024 and 2025 in treating the facts as human rights violations attributable to the State. In December 2025 the Supreme Court adopted the Inter-American Court’s category of serious human rights violation (CEDAW Committee, 2024, para. 8.6; Inter-American Court, 2025, para. 225; Supreme Court of Justice of Peru, 2025). The Constitutional Court, however, read Law 32107 in another direction (Constitutional Court of Peru, 2025, legal ground 210). The Inter-American Court’s judgment and the Supreme Court’s order for a new trial are binding, but their implementation was still pending as of September 2026, while Law 32107 remained in force and statutory limitation remained in dispute (Inter-American Court, 2025, operative paragraph 8; Supreme Court of Justice of Peru, 2025, decision III).

Table 1 records the result for each of the four propositions. P1 to P3 are supported by the documents and decisions examined in Sections 5 and 6. None of the refutation conditions stated in Section 4 is met, since the instruments presented the program as voluntary, the facts did not keep a single characterization, and later decisions reasoned from those instruments. P4 cannot be confirmed by one case, but its outcomes are consistent with it. Accountability appeared at the level of the State, where official bodies agreed and binding decisions followed, and it has not appeared at the level of individuals, where official bodies disagreed and statutory limitation remained in dispute.

The case also adds a caveat to the regional trend toward accountability. Roht-Arriaza (2015) shows that amnesties, statutes of limitation and the absence of international crimes from domestic codes, which for decades kept cases out of court, have fallen as barriers in most Latin American countries. She also describes this change as fragile. In Peru, Fujimori’s conviction for Barrios Altos and La Cantuta was followed by a humanitarian pardon in December 2017 (Inter-American Court, 2025, para. 94). A Supreme Court judge declared the pardon devoid of legal effect in 2018, but the Constitutional Court restored it in 2022 and in December 2023 ordered Fujimori’s immediate release. The Inter-American Court, which had ordered the State not to implement those decisions, then declared the State in contempt (Inter-American Court, 2026, paras. 8, 11, 47, operative paragraph 2). Law 32107 restores statutory limitation for facts prior to 2002, and the Constitutional Court’s call on Congress to incorporate crimes against humanity into the Criminal Code shows that the domestic code lacked that definition in 2025 (Law 32107, 2024, art. 4; Constitutional Court of Peru, 2025, operative paragraph 3). In August 2025 a new amnesty law was promulgated for members of the armed forces, the police and self-defense committees, covering acts committed between 1980 and 2000 (Law 32419, 2025). The Inter-American Court ordered the State not to apply it, and the executive called that order unacceptable (LP, 2025; Xinhua, 2025). Lutz and Sikkink (2001) distinguish a shift in norms from a change in behavior, and the case fits that distinction: international norms on the sterilizations advanced while domestic legislation moved in the opposite direction.

The characterization of the facts matters even where no conviction follows. In her concurring opinion, Judge Hernández López argued that characterizing the facts as a crime against humanity would not attribute individual criminal responsibility but would recognize the nature and gravity of the violation (Inter-American Court, 2025, concurring opinion of Judge Hernández López, para. 48). Given Law 32107, she added, “to name the facts legally according to their true nature is a minimum requirement of truth and justice” (Inter-American Court, 2025, concurring opinion of Judge Hernández López, para. 49; our translation). Recognition, the responsibility of the State and reparation are forms of accountability in their own right, and deferred impunity refers only to the part of accountability that remains unmet.

Because these conclusions rest on one national case and one comparison within it, the conditions stated here are hypotheses for comparative work, not general findings. Programs whose first phase resembles the Peruvian one but whose second phase took a different course would test them, and so would cases in which a single decision settled statutory limitation. The Peruvian second phase is also still open. As of September 2026, no information on the new trial ordered in December 2025 was available, and the two highest domestic courts disagreed on statutory limitation. The argument concerns the conditions under which a characterization leads to the attribution of responsibility, and it says nothing about the guilt or innocence of any person.

## 8 Conclusion

This article asked what role law played in the forced sterilizations carried out in Peru, both while the program operated and in the decades that followed. Between 1996 and 2000 the sterilizations were authorized and recorded through a law, a ministerial resolution, numerical sterilization targets and consent forms. From 1,997 onward, prosecutors, Congress, the executive, domestic courts and international bodies gave the same facts incompatible legal characterizations. Existing concepts described either the legitimation of the program or the later dispute over it, but not the connection between the two.

We proposed the juridical laundering of biopower to name that connection. The category describes a process that works through legal documents and unfolds in two phases. In the first phase, legally valid instruments present a state intervention in the life, health or reproduction of a population as lawful, technical and, where it is framed as voluntary, consented to. In the second phase, official bodies of the same juridical field assign successive and incompatible legal characterizations to that intervention, and the characterization that prevails at any given moment determines whether responsibility can be attributed. Four moments give the category its content: legal inscription, technical neutralization, consent as sign and deferred recharacterization.

The Peruvian case shows both phases and the link between them. At the time of the program, a law and a ministerial resolution gave it a lawful purpose, expressed in the language of rights and in that of demographic goals. Targets and quotas turned it into a matter of coverage, and signed forms recorded each operation as the woman’s own decision. Afterwards, the same facts received at least six legal characterizations, from a fortuitous event to a serious human rights violation, and no court adopted the characterization of crime against humanity. Twice, prosecutors declined to charge the officials who had designed the program on grounds taken from the program’s own instruments.

Our position is that the legal documents that made the program look legitimate kept having effects after it ended. The instruments that legitimized it at the time later supplied evidence and arguments to those who resisted a new characterization, which is what we call the long temporality of the symbolic efficacy of law. Recharacterization led to accountability where different official bodies agreed on the new legal characterization, a binding decision enforced it, and the rules on statutory limitation allowed prosecution. Where these conditions failed, recharacterization led instead to deferred impunity, a prolonged failure to hold any individual criminally responsible. In Peru both outcomes coexist: the responsibility of the State has been acknowledged and declared, while no official who designed or directed the program has been brought to trial. The former president under whom it was carried out died in 2024 without having been tried for the sterilizations.

The category offers the sociology of law an account of how legal form continues to operate after the events it authorized. It distinguishes violence carried out through ordinary law from violence shielded afterwards by an amnesty. An amnesty can be undone by a single decision, whereas violence carried out through ordinary law must be contested case by case, against documents that record it as lawful and consented to. For the study of accountability, the case indicates that the fall of amnesties does not end the matter, because a new statute can restore statutory limitation or grant a new amnesty, as the laws of 2024 and 2025 show.

These conclusions rest on one national case and on one comparison within it, and the second phase of the Peruvian case remained open as of September 2026. The conditions we have stated are therefore hypotheses. Programs that share the first phase of the Peruvian one, such as those documented in Central Europe and North America, would allow these hypotheses to be tested against second phases that took different courses. The category can also be examined beyond reproductive policy, in other state interventions in the life of populations that were carried out through legal instruments. In each case the question is the one this article has asked: how law made violence appear legitimate when it authorized it, and how it characterized that violence afterwards.

## Data Availability

The original contributions presented in the study are included in the article, further inquiries can be directed to the corresponding author.
